# Experiências de iniciação sexual e desigualdades de gênero entre adolescentes em um bairro de alta vulnerabilidade social em Salvador, Bahia, Brasil

**DOI:** 10.1590/0102-311XPT129925

**Published:** 2026-03-09

**Authors:** Manuela Maciel Souza Codeço, Simone Souza Monteiro, Andrea Fachel Leal, André Luiz Machado das Neves, Regina Maria Barbosa, Naiara Maria Santana Neves, Daniela Riva Knauth, Laio Magno

**Affiliations:** 1 Departamento de Ciências da Vida, Universidade do Estado da Bahia, Salvador, Brasil.; 2 Secretaria Municipal de Saúde, Prefeitura Municipal de Salvador, Salvador, Brasil.; 3 Instituto Oswaldo Cruz, Fundação Oswaldo Cruz, Rio de Janeiro, Brasil.; 4 Instituto de Filosofia e Ciências Humanas, Universidade Federal do Rio Grande do Sul, Porto Alegre, Brasil.; 5 Escola Superior de Ciências da Saúde, Universidade do Estado do Amazonas, Manaus, Brasil.; 6 Núcleo de Estudos de População Elza Berquó, Universidade Estadual de Campinas, Campinas, Brasil.; 7 Departamento de Filosofia e Ciências Humanas, Universidade Estadual de Santa Cruz, Ilhéus, Brasil.; 8 Faculdade de Medicina, Universidade Federal do Rio Grande do Sul, Porto Alegre, Brasil.; 9 Instituto Gonçalo Moniz, Fundação Oswaldo Cruz, Salvador, Brasil.

**Keywords:** Sexualidade, Saúde do Adolescente, Perspectiva de Gênero, Saúde Sexual e Reprodutiva, Infecções Sexualmente Transmissíveis, Sexuality, Adolescent Health, Gender Perspective, Reproductive Health, Sexually Transmitted Diseases, Sexualidad, Salud del Adolescente, Perspectiva de Género, Salud Sexual y Reproductiva, Enfermedades de Transmisión Sexual

## Abstract

A adolescência é um período marcado por descobertas e vivências sexuais, frequentemente atravessado por normas e relações de gênero que produzem hierarquias e desigualdades. O objetivo deste estudo é compreender as experiências de iniciação sexual de adolescentes de um bairro popular de Salvador, Bahia, Brasil, sob a perspectiva de gênero. Trata-se de pesquisa qualitativa, recorte do estudo multicêntrico *Contextos de Vulnerabilidade ao HIV entre Jovens de Camadas Populares (Projeto Espaços Jovens)* realizado em cinco capitais brasileiras. Foram analisadas 36 entrevistas individuais e quatro grupos naturais conduzidos em Salvador entre maio de 2021 e maio de 2022. O material empírico foi submetido à análise de conteúdo temática, com foco nas categorias: gênero, iniciação sexual, práticas sexuais e contexto familiar. As experiências de iniciação sexual mostraram-se diversas e influenciadas por marcadores sociais, especialmente o gênero. Enquanto as meninas relataram maior preocupação com julgamentos sociais e idealizações afetivas, os meninos vivenciaram a sexualidade com mais liberdade, reforçando expectativas da masculinidade hegemônica. As barreiras ao diálogo com as famílias limitaram o acesso a informações qualificadas sobre saúde sexual e reprodutiva. As desigualdades de gênero estruturaram as vivências sexuais na adolescência, contribuindo para a ampliação de vulnerabilidades e a limitação do acesso à educação sexual. Os achados reforçaram a necessidade de políticas públicas intersetoriais que promovam uma abordagem emancipatória da sexualidade, com foco em direitos, diversidade e equidade.

## Abstract (en)

Adolescence is a period marked by sexual discoveries and experiences, often influenced by gender norms and relations that produce hierarchies and inequalities. The aim of this study is to understand experiences of sexual initiation among adolescents in a low-income neighborhood in the city of Salvador, Bahia State, Brazil, from a gender perspective. A qualitative study was performed using data from the multicenter study entitled *Contexts of Vulnerability to HIV among Young People from Lower Classes (Youth Spaces Project)* conducted in five Brazilian capitals. Thirty-six individual interviews and four natural groups conducted in Salvador between May 2021 and May 2022 were analyzed. Empirical material was submitted to thematic content analysis, focusing on the categories gender, sexual initiation, sexual practices, and family context. The experiences of sexual initiation were diverse and influenced by social markers, especially gender. While girls reported greater concern with social judgments and emotional idealizations, boys experienced sexuality more freely, reinforcing expectations of hegemonic masculinity. Barriers to dialogue with the family limited access to qualified information on sexual and reproductive health. Gender inequalities structured sexual experiences in adolescence, contributing to the expansion of vulnerabilities and the limitation of access to sex education. The findings underscore the need for intersectoral public policies that promote an emancipatory approach to sexuality, with a focus on rights, diversity, and equity.

## Abstract (es)

La adolescencia es un período marcado por descubrimientos y experiencias sexuales, a menudo atravesado por normas y relaciones de género que producen jerarquías y desigualdades. El objetivo de este estudio es comprender las experiencias de iniciación sexual de adolescentes de un barrio popular de Salvador, Bahía, Brasil, desde una perspectiva de género. Se trata de una investigación cualitativa, recorte del estudio multicéntrico *Contextos de Vulnerabilidad al VIH entre Jóvenes de Estratos Populares (Projeto Espaços Jovens)*, realizado en cinco capitales brasileñas. Se analizaron 36 entrevistas individuales y cuatro grupos naturales realizados en Salvador entre mayo del 2021 y mayo del 2022. El material empírico se sometió a análisis de contenido temático, centrándose en las categorías: género, iniciación sexual, prácticas sexuales y contexto familiar. Las experiencias de iniciación sexual han demostrado ser diversas y estar influenciadas por marcadores sociales, especialmente el género. Mientras que las chicas relataron una mayor preocupación por los juicios sociales y las idealizaciones afectivas, los chicos experimentaron la sexualidad con más libertad, lo que reforzó las expectativas de masculinidad hegemónica. Las barreras para el diálogo con las familias limitan el acceso a información calificada sobre salud sexual y reproductiva. Las desigualdades de género moldean las experiencias sexuales durante la adolescencia y contribuyen para aumentar las vulnerabilidades y la limitación del acceso a la educación sexual. Los hallazgos reforzaron la necesidad de políticas públicas intersectoriales que promuevan un enfoque emancipador de la sexualidad, centrándose en los derechos, la diversidad y la equidad.

## Introdução

A adolescência é geralmente o período da vida em que ocorrem experiências de iniciação sexual ^1^ ^,^ ^2^, que permitem aos(às) adolescentes a realização de novas descobertas e experimentações ^3^ ^,^ ^4^. Embora a adolescência seja abordada do ponto de vista cronológico, não há consenso sobre quando de fato essa fase da vida se inicia ou mesmo finaliza. A Organização Mundial da Saúde (OMS) e a Academia Americana de Pediatria definem que a adolescência compreende o período de 10 a 19 anos e 11 a 21 anos, respectivamente ^5^ ^,^ ^6^. Essas diferenças mostram como a adolescência não pode ser fixada como um fenômeno natural e nem mesmo universal. Estudos socioantropológicos têm apontado a adolescência como uma construção social e dependente dos contextos socioeconômicos e culturais nos quais cada sujeito está inserido ^7^.

Apesar disso, a concepção de adolescência nas pesquisas e práticas de cuidado em saúde sexual tem sido interpretada predominantemente com base em termos essencialistas (uma interpretação de fenômenos socioculturais como naturais) e universal (generalização baseada em uma série de simplificações e apagamentos de cunho temporal, social e cultural) ^7^. Sob esta perspectiva, a adolescência é geralmente vista como um período complexo e problemático ^8^ ^,^ ^9^.

Na contramão desse entendimento, podemos lançar mão de uma visão fenomenológica das experiências de adolescer, que podem ser compreendidas como uma abertura para o mundo ^7^. Nessa perspectiva, as experiências vivenciadas pelos(as) adolescentes são compreendidas como um caleidoscópio de significações, que se transformam de acordo com as interações intersubjetivas. Desse modo, não podemos deixar de destacar que essas experiências são intrinsecamente articuladas com aspectos de classe social, raça/cor de pele, gênero e nível educacional e outros marcadores de diferença social ^9^ ^,^ ^10^.

Nesse sentido, compreendemos que as experiências de iniciação sexual de adolescentes afetam a saúde sexual e reprodutiva. Estudos demonstram que a primeira relação sexual genital na adolescência - definida como aquela que envolve penetração vaginal ou anal - pode estar associada a diferentes desfechos, como gravidez não planejada e aborto inseguro, no caso das relações vaginais, e a infecções sexualmente transmissíveis (IST) e julgamento social negativo em ambos os tipos de prática sexual (i.e., vaginal ou anal) ^11^ ^,^ ^12^. Além disso, pode produzir efeitos mais deletérios para as mulheres, apontando para atravessamentos de gênero dessa experiência ^11^. Por outro lado, a maternidade pode também assumir significados simbólicos positivos em contextos de vulnerabilidade socioeconômica, sendo às vezes percebida como um presente divino, fonte de amor e apoio futuro e fortalecimento de vínculos sociais e afetivos ^13^. Esses desfechos podem ocorrer especialmente quando a primeira relação sexual genital acontece sem apoio social e/ou sem o acesso à informação adequada ^14^.

Apesar dessa realidade, a resposta de serviços de saúde para a promoção de saúde sexual e reprodutiva tem sido limitada. É comum encontrar profissionais de saúde que ainda adotam a prática biomédica tradicional, fundamentada apenas em uma perspectiva de risco epidemiológico, sem considerar aspectos subjetivos do cuidado ^15^, e que pode limitar o escopo do olhar em torno da iniciação sexual na adolescência e prejudicar as possibilidades de intervenções mais ampliadas que considerem as necessidades de saúde sexual e reprodutiva ^16^.

Além disso, essa perspectiva pode reforçar estigmatização em torno da iniciação sexual, ao associá-la essencialmente à ideia de risco, diminuindo as noções de autonomia, prazer, afetividade e descoberta. Essa prática pode afastar os(as) adolescentes dos serviços de saúde, que não têm sido espaços para promoção de saúde sexual e reprodutiva ^17^. Desse modo, este estudo objetiva compreender as experiências de iniciação sexual de adolescentes de um bairro popular de Salvador, Bahia, Brasil, sob a perspectiva de gênero.

## Métodos

Trata-se de estudo qualitativo com abordagem fenomenológica, integrado à pesquisa guarda-chuva intitulada *Contextos de Vulnerabilidade ao HIV entre Jovens de Camadas Populares: Um Estudo Multicêntrico em Cinco Cidades do Brasil (Projeto Espaços Jovens)*, que analisou contextos de exposição ao HIV e outras IST entre adolescentes e jovens com idade de 15 a 19 anos, moradores de comunidades de baixa renda, localizadas em Porto Alegre (Rio Grande do Sul), São Paulo, Salvador, Manaus (Amazonas) e Rio de Janeiro. Para mais informações, o papel das interações entre marcadores sociais de raça/cor, gênero e classe social nas relações sociais e afetivas das adolescentes mulheres de todos os sítios foi analisado em outro artigo ^18^.

Neste artigo, fruto da dissertação de mestrado de M.M.S.C. ^19^, analisamos as entrevistas individuais e grupos naturais realizados com adolescentes de 15 a 19 anos, moradores de um bairro popular da cidade de Salvador. Com aproximadamente 17 mil pessoas, majoritariamente jovens, mulheres e negros ^20^, esse território é historicamente habitado por uma população remanescente de quilombo ^21^ ^,^ ^22^, que vive em um contexto de alta vulnerabilidade social ^23^ e exposta à violência do Estado e ao racismo ^21^.

O trabalho de campo foi feito entre maio de 2021 e maio de 2022, por três mulheres e um homem cisgêneros (cis) com experiência em pesquisa social. Foram realizadas 36 entrevistas individuais com meninos cis, meninas cis e transgênero (trans), com duração média de 47 minutos cada, com perguntas disparadoras sobre experiências, conhecimentos e práticas de prevenção e de risco relativos à saúde sexual e reprodutiva. A quantidade mínima de entrevistas (n = 30) em cada sítio de pesquisa foi definida *a priori*. Entretanto, em Salvador, foi observada a necessidade de ampliação do número estabelecido para dar conta do critério de saturação deste subestudo ^24^.

Neste bairro, foram mapeados espaços de socialização da comunidade, que serviram como pontos estratégicos de aproximação com os participantes para captação *in situ* para a pesquisa. A interação com os(as) adolescentes ocorreu de forma respeitosa e acolhedora, embora tenha sido percebida de maneira distinta pelos(as) participantes. Alguns relataram iniciar as entrevistas com certa timidez, a qual tendia a diminuir à medida que o diálogo avançava e se estabelecia uma relação de confiança com os(as) entrevistadores(as). Também foram utilizados informantes-chave na comunidade - profissionais de saúde, especialmente agentes comunitários de saúde e lideranças comunitárias - para indicação de adolescentes elegíveis para a pesquisa. Esses indicaram outros(as) adolescentes.

Os quatro grupos de jovens foram realizados em uma escola da comunidade, devido à facilidade do acesso e à disponibilidade de espaço. Os alunos foram agrupados por série/turma e totalizaram 46 participantes, com duração média de 80 minutos. Os grupos eram compostos por meninos cis e meninas cis, de faixa etária próxima, e, em sua maioria, não eram os mesmos participantes das entrevistas individuais. Cada grupo foi conduzido por um moderador e outras pessoas integrantes do projeto, que colaboraram no apoio às atividades, observação e registro. Foram utilizados gravadores, um roteiro com perguntas disparadoras e um mapa para localização dos integrantes.

As entrevistas e falas registradas nos grupos naturais foram transcritas por pesquisadores do projeto, categorizadas, codificadas e analisadas com base na análise de conteúdo temática ^25^. Tal processo resultou nas seguintes categorias temáticas: experiências de iniciação sexual, significados da virgindade, motivações e pressão social e insuficiente apoio familiar. A interpretação dos achados foi orientada pela revisão da literatura e pelos objetivos do estudo. Para preservar a identidade dos(as) adolescentes, foi utilizada a letra “E” para o(a) entrevistado e as letras “GN” para as falas ocorridas nos grupos naturais, seguida de numeral para identificar sequência numérica, idade da pessoa e as letras “F” para feminino, “M” para masculino e “MT” para mulher transsexual.

O projeto guarda-chuva foi submetido e aprovado pelo Comitê de Ética em Pesquisa (CEP) da Universidade do Estado da Bahia (parecer nº 4.540.024). Todas as pessoas participantes com idade igual ou superior a 18 anos assinaram o Termo de Consentimento Livre e Esclarecido (TCLE). Os menores de 18 anos assinaram o Termo de Assentimento Livre e Esclarecido, havendo parecer favorável do CEP para dispensa de TCLE para pais ou responsáveis nestes casos.

## Resultados e discussão

### Características gerais dos(as) participantes

Como indica a Tabela 1, dos(as) 36 entrevistados(as), um terço disse morar apenas com a mãe (12). A maioria era composta por meninas cis (19), com idades entre 15 e 17 anos, autodeclaradas pretas(os) (21) e heterossexuais (30), que afirmaram não estar namorando no momento (26). Grande parte declarou estar apenas estudando (20), no Ensino Fundamental (17), porém com distorção idade-série superior a dois anos (19). Quase a metade das mães desses jovens estudou até o Ensino Fundamental, mas não o completou (16) (Tabela 1).

**Tabela 1:** Características da população estudada. Salvador, Bahia, Brasil, 2022 (n = 36).

Variáveis	n	%	Mulher cisgênero	Mulher transgênero	Homem cisgênero
n (%)	n (%)	n (%)
Sexo atribuído ao nascer					
Feminino	19	52,8	19 (52,8)		
Masculino	17	47,3		3 (8,3)	14 (38,9)
Idade (anos)					
15	8	22,2	5 (26,3)	-	3 (21,4)
16	7	19,5	6 (31,6)	-	1 (7,1)
17	6	16,7	5 (26,3)	-	1 (7,1)
18	9	25,0	2 (10,5)	2 (66,7)	5 (35,7)
19	6	16,7	1 (5,3)	1 (33,3)	4 (28,6)
Raça/Cor					
Preta	26	72,2	13 (68,4)	3 (100,0)	10 (71,4)
Parda	9	25,0	5 (26,3)	-	4 (28,6)
Branca	1	2,8	1 (5,3)	-	-
Orientação sexual					
Heterossexual	30	83,4	17 (89,4)	2 (66,7)	11 (78,6)
Homossexual	2	5,6	1 (5,3)	-	1 (7,1)
Bissexual	1	2,8	1 (5,3)	-	-
Pansexual	1	2,8	-	-	1 (7,1)
Não se define	2	5,6	-	1 (33,3)	1 (7,1)
Tem namorado(a)					
Sim	10	27,8	9 (47,4)	-	1 (7,1)
Não	26	72,2	10 (52,6)	3 (100,0)	13 (92,9)
Ocupação					
Só estuda	20	55,5	14 (73,7)	1 (33,3)	5 (35,7)
Só trabalha	2	5,6	2 (10,5)	-	-
Trabalha e estuda	9	25,0	2 (10,5)	2 (66,7)	5 (35,7)
Desempregado	2	5,6	-	-	2 (14,3)
Nem trabalha, nem estuda	3	8,3	1 (5,3)	-	2 (14,3)
Escolaridade					
Ensino Fundamental incompleto	17	47,2	9 (47,4)	2 (66,7)	6 (42,8)
Ensino Médio incompleto	16	44,5	9 (47,4)	1 (33,3)	6 (42,8)
Ensino Médio completo	3	8,3	1 (5,2)	-	2 (14,3)
Distorção idade-série					
Sem atraso	8	22,2	5 (26,3)	-	3 (21,4)
Atraso de 1 ano	9	25,0	7 (36,8)	-	2 (41,3)
Atraso de 2 anos	8	22,2	3 (15,8)	-	5 (35,8)
Atraso de 3 anos	3	8,4	1 (5,2)	1 (33,3)	1 (7,1)
Atraso de 4 anos ou mais	8	22,2	3 (15,8)	2 (66,7)	3 (21,4)
Escolaridade materna					
Ensino Fundamental incompleto	16	44,4	7 (36,8)	2 (66,7)	7 (50,0)
Ensino Médio incompleto	8	22,2	6 (31,6)	1 (33,3)	1 (7,1)
Ensino Médio completo	6	16,7	4 (21,1)	-	2 (14,3)
Não sabe informar	6	16,7	2 (10,5)	-	4 (28,6)
Com quem mora					
Com a mãe	12	33,3	5 (26,3)	1 (33,3)	2 (14,3)
Com mãe e pai	9	25,0	4 (21,1)	1 (33,3)	4 (28,6)
Com mãe e padrasto	4	11,1	2 (10,5)	-	2 (14,3)
Com pai e madrasta	2	5,5	1 (5,3)	-	1 (7,1)
Com avós	5	13,9	1 (5,3)	-	4 (28,6)
Sozinha	3	8,3	1 (5,3)	1 (33,3)	1 (7,1)
Com outros familiares	1	2,8	1 (5,3)	-	-

Fonte: elaboração própria.

### Experiências de iniciação sexual

A maioria (32) dos(as) adolescentes declarou ter tido pelo menos uma relação sexual genital com idade inferior a 15 anos. Inquérito escolar realizado no Rio de Janeiro, entre 2016 e 2017, com adolescentes e jovens de 15 a 24 anos, mostrou que as meninas cis apresentaram maior proporção de iniciação sexual com idade entre 10 e 14 anos, sendo que aquelas mais pobres e de escolas públicas tiveram maior proporção em comparação com as mais ricas e de escolas privadas ^26^.

No nosso estudo, as diversas experimentações sexuais ocorreram em meio a descobertas, curiosidades e sentimentos, que podem se estender do primeiro acesso à pornografia, ao primeiro beijo e à primeira relação sexual genital. Para alguns(mas), essas experiências ocorreram precocemente, quando eram “*jovens demais*” ou “*crianças*”, em momentos marcados pela ingenuidade e brincadeiras que resultaram em contato sexual.

“*Primeira vez que eu fui, eu era criança, porque tava eu, a amiga de minha prima e minha prima. Aí elas ‘ah vamos brincar de pai e filha?’ Eu também, pra não dar um de besta, bora*” (E12-M-15 anos).

“*Antigamente, quando eu tinha 15, 16 anos, não sabia das coisas. Eu fazia muito uso da pornografia. Aí, quando foi na pele, foi totalmente diferente* (...) *Eu me cobro muito, porque eu gosto de satisfazer a pessoa*” (E28-M-18 anos).

As experiências de iniciação sexual são atravessadas por relações e normas de gênero, as quais produzem hierarquias baseadas na distribuição desigual do poder entre homens e mulheres ^11^. Essas normas reforçam expectativas socioculturais sobre performances masculinas e femininas, que podem silenciar e reprimir tanto homens quanto mulheres (sejam cisgênero ou transgênero) ^27^ ^,^ ^28^ ^,^ ^29^. A perspectiva de gênero mostra, portanto, que essas diferenças não são determinadas biologicamente, mas partem de construção sociocultural que perpetua desigualdades de poder ^30^ ^,^ ^31^ ^,^ ^32^.

Essas normas de gênero se reproduzem entre jovens de diferentes classes sociais, embora assumam formas distintas conforme o contexto. Em estudo realizado em Belo Horizonte (Minas Gerais) entre 2008 e 2009, Chacham & Jayme ^33^ observaram que, nas classes populares, tais normas se manifestam de modo mais explícito, frequentemente associadas ao controle masculino e à violência física ou simbólica nas relações afetivo-sexuais. Já entre jovens de classe média, tendem a se expressar de forma mais sutil, por meio de expectativas simbólicas de comportamento e papéis sociais. Ainda assim, os efeitos dessas normas são desiguais, resultando em maior vulnerabilidade para as meninas cis das camadas populares, que enfrentam restrições mais severas à autonomia sexual e reprodutiva.

Entre as meninas cis, houve relatos de planejamento da primeira relação sexual genital, com base na idealização acerca do tempo correto e da pessoa que correspondesse às expectativas de “*gosto*” e “*conexão*”.

“*A gente já tinha conversado, e eu disse a ele que ia esperar meu momento, e quando achasse que era meu momento aconteceria. E aí chegou o momento que eu achei que tava na hora e aconteceu*” (E31-F-17 anos).

Estudos prévios mostram que meninas cis geralmente decidem sobre a iniciação sexual baseando-se em normas de gênero que estimulam que o comportamento da mulher seja o de resistir inicialmente às investidas sexuais masculinas e ceder apenas posteriormente, visto que a iniciativa sexual é socialmente prerrogativa masculina ^34^ ^,^ ^35^. O encontro da “*pessoa certa*” e o tempo de relacionamento afetivo, por exemplo, são apontados por estudo realizado em Teresina (Piauí), em 2008, com adolescentes de 15 a 19 anos, como uma justificativa para o adiamento da primeira relação sexual com penetração ^36^.

Entretanto, neste estudo, algumas meninas cis não demonstraram esse perfil e referiram-se às experiências de iniciação sexual após experimentações com diversas pessoas, independentemente da orientação sexual, por desejo e/ou curiosidade.

“*A primeira vez com homem foi com 14 anos e com mulher, com 16. Mas, desde os meus 12 anos, eu já tinha atração por mulheres. Eu escondia, porque tinha vergonha de contar, até hoje meus pais não sabem*” (E18-F-18 anos).

“*Minha prima tem 18 anos e ainda tá na espera de um menino certo pra ela perder a virgindade. Isso é uma idiotice, porque não existe homem certo*” (GN1-F).

Nesse sentido, há uma ambivalência entre o desejo de viver uma sexualidade livre e as normas sociais de gênero. Ao mesmo tempo em que houve expressão em favor de maior liberdade sexual, também houve destaque para o medo de julgamentos. Essa postura de liberdade sexual pode refletir no processo de desconstrução de normas de gênero impostas às mulheres, fruto das contribuições históricas dos movimentos feministas ^37^.

Entre os meninos cis, as experiências de iniciação sexual foram relatadas com menos preocupação e mais abertas à experimentação. Todavia, isso não se dava sem pressão social e cobranças, como, por exemplo, necessidade de “*satisfazer a pessoa*”.

Entre meninas trans (3), por exemplo, as fronteiras borradas de identidade de gênero e orientação sexual foram marcantes no processo de descobrimento da sexualidade e na iniciação sexual. Este fato é confirmado nas experiências relatadas, num processo de reconhecimento e afirmação da performance de gênero feminina que ocorre no transcorrer da adolescência.

“*A minha primeira experiência sexual, eu ia fazer 14 anos. Pouco antes de me assumir, eu lembro que eu tava na dúvida ainda, foi com um menino que eu gostava dele, ele também gostava de mim e eu gostei* (...) *no caso eu era gay, eu fui o primeiro gay que ele pegou. Ele foi o primeiro menino*” (E36-MT-18 anos).

Desse modo, experiências de iniciação sexual podem ser consideradas como uma abertura para um mundo de novas experimentações, que se dão em articulação íntima entre normas de gênero e orientações sexuais/performances de gênero ^38^ ^,^ ^39^. Essas experiências não são limitadas às práticas sexuais genitais, como quase sempre a perspectiva biomédica tem postulado, mas, envolvem relações intersubjetivas com a produção de afeto, desejos, preferências e intimidade, que se transformam no horizonte do encontro entre pares ^39^ ^,^ ^40^.

### Significados da virgindade

A virgindade foi significada pelo fenômeno da primeira relação sexual genital, desconsiderando experiências sexuais não genitais. Entre aqueles(as) que não vivenciaram uma prática sexual genital, a escolha pelo adiamento decorreu do reconhecimento do desejo de encontrar o “*momento*” ou a “*pessoa certa*” para “*se entregar*”, especialmente entre meninas cis.

“*Claro que você não vai se entregar pra qualquer pessoa. Claro que não!*” (E26-F-16 anos).

“*Eu perdi a virgindade com 14 anos, porque achei que era meu momento* (...) *Esperei o momento de tá num relacionamento*” (GN1-F).

Entre meninas cis e meninas trans, a virgindade foi objetificada como algo que deveria ser guardado para um momento especial e romântico, que é perdido no momento da penetração, vaginal ou anal, durante uma relação sexual genital. Nesses relatos, foi comum a experiência de dor e não de prazer, considerada uma situação normal de acordo com um conselho materno de uma das meninas cis. Esse elemento é fruto de uma construção social de gênero que normaliza a ideia de iniciação da vida sexual genital com dor e como sinônimo de perda, impureza e perigo de desvalorização social ^34^ ^,^ ^41^.

“*Ele foi meu primeiro homem. Foi com ele que eu perdi minha virgindade. A gente ficava só no beijo, aí depois que eu me entreguei a ele, a gente começou a namorar*” (E7-MT-18 anos).

“*Aconteceu* [primeira relação sexual]*. Não foi nada demais, não significou nada. Senti somente dor*” (E5-F-17 anos).

“*Minha mãe falava que doía um pouquinho. E que eu ia me acostumar, porque a dor era, ela falava, era relativo, e que ia sangrar um pouquinho, e que depois ia ser normal*” (E19-F-16 anos).

Entre meninas cis, a virgindade parece ter sido também apresentada como um marco importante de transição para a vida adulta, que exigiria mais “*responsabilidades*”, como a adoção de práticas de cuidado com o corpo e uso de métodos contraceptivos. A possibilidade de gestação não planejada, por exemplo, foi relatada como um evento de impacto negativo na experiência dessas adolescentes.

“*Com 13 anos de idade. Desde então, tive que criar responsabilidades, para não engravidar* (...) *minha mãe não ia aceitar, ia ser um transtorno na minha vida. Eu não teria como sustentar e educar essa criança, porque eu sou uma criança praticamente*” (E5-F-17 anos).

Entre meninos cis, a descrição das experiências de iniciação sexual e da primeira relação sexual genital aparece de maneira superficial, sem grande aprofundamento sobre sentimentos e afetos, com frases curtas, tais como “*foi de boa*”, “*normal*”, “*não sei nem o que dizer*”, possivelmente relacionadas com uma necessidade de se manter distanciado do tema, ou mesmo para não demonstrar emoções, ou por se sentirem desconfortáveis ou inseguros ao falar sobre sexualidade.

“*Eu acho que foi com 15* [anos]*. Foi de boa, na casa de minha tia*” (E33-M-19 anos).

“*Já tem muito tempo. Eu tinha 16 anos. Foi normal. Eu tava 1 ano já com essa menina, depois resolvi ter* [relação sexual]” (E34-M-19 anos).

Esses relatos masculinos podem indicar um comportamento que desfavorece o planejamento de relações sexuais protegidas, contribuindo para que os adolescentes tenham relação sexual “*sem tomar cuidado*”. Nos discursos, alguns podem não recusar relações sexuais com penetração na ausência de preservativo, contribuindo para situações de vulnerabilidade às IST e gravidez.

“*Nas vezes que foi uma rapidinha* (...) *num local que eu posso ser pego no flagra, foi sem tomar nenhum cuidado* [sem preservativo]” (E2-M-18 anos).

“*A gente vai, na intenção de usar camisinha. Na hora que tá se beijando, o fogo bate* (...) *Só tira de dentro quando terminar*” (GN1-M).

Observamos pressão e cobrança dos pares. As expectativas sociais sobre comportamentos sexuais masculinos estabelecem que homens devem ser assertivos, dominantes, sexualmente experimentados e bem-sucedidos ^27^ e, portanto, não devem mostrar-se frágeis diante de uma oportunidade de relação sexual ^42^. Essa pressão pode gerar ansiedade, insegurança e limitar a liberdade deles para expressão de emoções e de busca de ajuda em momentos de dificuldade ^28^. Nesse sentido, elementos de masculinidade hegemônica impõem aos meninos cis pressão social que pode ser prejudicial, como apontado por outros estudos ^42^ ^,^ ^43^ ^,^ ^44^.

### Motivações e pressão social

São várias as motivações que impulsionaram os(as) adolescentes na iniciação sexual, como, por exemplo, curiosidade, desejo de intimidade e conexão emocional, desejo de se encaixar em um grupo ou mesmo a pressão social do grupo. Alguns(mas) adolescentes demonstraram curiosidade em relação ao sexo e à sexualidade, ao “*querer saber*”, experimentar novas sensações e aprender mais sobre seus corpos e os dos parceiros. Nesse processo, também se dá a descoberta dos desejos e das preferências sexuais.

“*A primeira foi tranquila, doeu um pouco só. Foi por acaso. Não sei como, por mais incrível que pareça, foi lá dentro da escola mesmo, atrás da escola* (...) *Eu queria saber, assim, como era*” (E21-F-17 anos).

“*Eu fui descobrindo* [sobre a sexualidade] *com o tempo com ela. Ela também era virgem, aí fomos descobrindo juntos, foi ótimo* [viver esse momento juntos]*...*” (E28-M-18 anos).

As experiências de iniciação sexual podem significar momentos em que é possível expressar sentimentos, fortalecer relacionamentos e reforçar o desejo pelo prazer, intimidade e conexão emocional nas relações afetivas. Alguns(mas) participantes relacionaram as experiências em relações sexuais genitais a uma forma de expressar amor ou compromisso com o parceiro, mais comum entre meninas cis, enquanto outros(as) tenderam a vê-las como uma forma de atender ao prazer, geralmente entre meninos cis.

“*Ele foi um homem de verdade, que me aceitou, não tinha vergonha de nada. Tinham momentos que os amigos dele todos falavam, mas ele não ligava*” (E7-MT-18 anos).

“*No geral, o que pintar na rede é peixe. A gente não tem esse preconceito* (...) *a gente pensa mais no prazer mesmo, não tem mais aquela coisa linda de antigamente, de casar, ter filho, se apaixonar*” (E8-M-19 anos).

A necessidade de atender às expectativas dos seus semelhantes também foi relatada como motivação para o início das relações sexuais genitais entre meninas cis, na medida em que podem se sentir envergonhadas ou intimidadas em assumir a virgindade perante o grupo. Essa pressão social pode cooperar para o silenciamento, encobrimento da verdade sobre a virgindade ou mesmo relutância em expressar inseguranças e expectativas sobre o processo de iniciação sexual. Nesse sentido, a pressão social foi relatada como um dos motivos para a primeira relação sexual genital, até mesmo contra o próprio desejo individual.

“*As meninas falavam, eu ficava toda sem graça, não sabia nem o que falar, só ficava escutando e dando risada. Eu achava que era para ter logo relação sexual, porque minhas amigas já tinham. Já menti muitas vezes* [sobre ter tido uma relação sexual]” (E1-F-15 anos).

“*Tem menino também que dá uma de furão e quando vai ver ainda é virgem*” (GN1-F).

Essa pressão foi relatada como mais forte no contexto do “*namoro*” - relacionamentos estáveis, com maior compromisso e mais duradouros -, visto que a ocorrência de penetração em uma relação sexual significaria o estabelecimento de maior laço de intimidade entre os parceiros.

“*Antigamente, quando eu namorava, minha vida sexual era ativa, né? Agora eu terminei meu relacionamento*” (E14-F-15 anos).

“*Com meu namorado, que foi a primeira vez e até hoje é com ele. Nunca foi com outra pessoa*” (E31-F-17 anos).

“*Foi com 13 anos. Foi com meu namorado, meu ex namorado. Ficamos 2 anos juntos, depois dele eu só tive relações com três pessoas, só*” (E35-F-15 anos).

É importante destacar que as primeiras experiências sexuais dos meninos cis podem ocorrer de diferentes maneiras e em diferentes tipos de relacionamentos. Alguns relataram que aconteceram prioritariamente em relacionamentos ocasionais, com “*ficantes*”, amigas ou primas. Apenas um menino cis relatou experiência de uma primeira relação sexual genital com uma namorada e um outro estava namorando no período de realização da entrevista. Este último ponderou que se tratava de relacionamento sério e mantinham uma relação sexual não genital, pois estavam aguardando o momento ideal.

“*Eu tinha 13 anos, 13 para 14 anos, comecei e foi com uma prima minha, que acho que tinha 16*” (E11-M-15 anos).

Houve o relato de diversas expectativas ligadas às relações afetivas, que variaram de acordo com as experiências e vivências pessoais dos(as) adolescentes. Algumas delas estavam relacionadas à expectativa frustrada do encontro com uma “*pessoa especial*”, que é desejada e sonhada. Outras estavam associadas à busca por um relacionamento estável, para expressar a carência de quem não quer mais apenas “*ficar*”, destaque declarado por um dos meninos cis.

“*Eu não sei se foi a pessoa ou pelo fato de não ser como eu imaginaria, com uma pessoa especial. Tem umas pessoas que a gente dá tudo ali né, imagina ‘ah vai ser foda’, mas não foi, não foi aquilo que a gente imaginou*” (E25-F-17 anos).

“*Senti* [vontade de ter relação sexual]*, mas, quando isso aconteceu, a pessoa só queria ficar comigo e nada mais. Havia interesse da minha parte, mas por parte da pessoa não* (...) *Tem momentos que eu fico carente*” (E10-M-18 anos).

Para muitos meninos cis, a atração física e o desejo de perder a virgindade são motivações centrais. No entanto, há uma diversidade de experiências, com alguns(mas) adolescentes adiando a iniciação sexual por razões românticas, como a busca por um relacionamento significativo. Essa dualidade entre desejo e afetividade reflete padrões sociais de masculinidade que, ao mesmo tempo que incentivam a experimentação, também valorizam o afeto ^45^.

### Insuficiente apoio familiar

O diálogo entre pais e adolescentes foi relatado como inexistente para alguns. Há uma queixa da falta de abertura dos pais para abordar questões e dúvidas relacionadas à vivência da sexualidade. Alguns(mas) adolescentes relataram falta de orientação e ter que iniciar a vida sexual às escondidas do conhecimento da família.

“*Quando eu preciso, ninguém nunca tá comigo. Às vezes, o que eu preciso nem é muito, é só conversar mesmo. Não consigo conversar com meus pais. Eu prefiro guardar a maioria das coisas pra mim*” (E15-F-15 anos).

“*Aí esse povo fica meio perdido, assim como eu também já fiquei perdido, até porque não tinha ninguém pra conversar, ninguém pra eu chegar e ter esse tipo de conversa. Eu fazia tudo escondido*” (E2-M-18 anos).

Apesar de haver relatos de relações dialógicas entre mães e filhas, que foram comparadas como uma relação entre amigas, observamos um limite sobre os assuntos abordados, existindo uma barreira que não permitia a aceitação da sexualidade dessas adolescentes.

“*Ela* [mãe] *é o tipo de pessoa que posso confiar* (...) *ela é minha melhor amiga* (...) *Mas minha mãe nunca conversou comigo sobre sexo, sobre meninos, nem nada disso* (...) *Foram eles que descobriram* [acharam receita de anticoncepcional] *e ficaram me questionando. Aí eu tive que contar*” (E1-F-15 anos).

Esse distanciamento da família pode impactar no estabelecimento de um espaço de diálogo sobre saúde sexual e reprodutiva. Estudos indicam que estratégias educativas baseadas no vínculo e na escuta ativa podem ajudar adolescentes a encontrar espaços de conversa para construir conhecimento sobre sua saúde ^46^. Mesmo entre aqueles(as) adolescentes que relataram conversar com os pais, observamos que as orientações sobre saúde sexual e reprodutiva eram superficiais e limitadas ao uso de preservativo e contraceptivo, como também observado em estudos realizados em outros países de baixa e média renda ^47^.

“*Ela fala pra eu usar camisinha, para eu tomar cuidado*” (E13-M-15 anos).

“*Então ela* [mãe] *nunca tocou no assunto, mas sempre falou: ‘Ah tem que usar camisinha’ pra se prevenir, tomar injeção, tudo certinho*” (E25-F-17 anos).

De modo geral, observamos a tentativa de maior controle dos corpos das meninas cis do que dos meninos cis pela família. Por exemplo, a “*virgindade*” é controlada e vista como algo importante para pais e outros familiares que exercem poder sobre essas meninas cis. Constantemente, as meninas cis são questionadas sobre a virgindade, como se fosse um ponto primordial, supervalorizando esse momento de “*perda*” e consequente conflito no núcleo familiar. Muitas adolescentes acabam omitindo as experiências de iniciação sexual por medo da aceitação familiar.

“*Eu tive que contar a ele que eu perdi minha virgindade* (...) *aí ele falou um monte de coisa. Minha mãe já foi dizendo que ia me mandar embora, para morar com meu tio. Aí ele perguntou como foi, aí eu falei ‘foi bom’, aí ele foi e me bateu*” (E1-F-15 anos).

“*Quando eu perdi a virgindade, contei pra minha mãe no mesmo dia, só que ela me tratou com ignorância*” (E24-F-18 anos).

Além disso, o preconceito quanto às orientações sexuais e identidades de gênero distintas da norma heterossexual também são um desafio que se apresenta às adolescentes bissexuais e meninas trans, para vivenciarem essas experiências na iniciação sexual. Essa falta de acolhimento parece catapultar algumas delas para viverem suas experiências sexuais de forma escondida.

“*Eu escondia* [a bissexualidade]*, porque tinha vergonha de contar. Meus pais não sabem, não sei nem como eu vou contar. Porque minha família tem muito preconceito*” (E18-F-18 anos).

“*Era uma relação de namoro escondido, porque meus pais nunca foram de aceitar* (...) *Com eles também não tem muita conversa. Meu pai, eu não me dou muito com ele* [não aceita a identidade de gênero] (...) *nunca tive coragem de levar ele* [namorado] *lá pra conhecer meus pais*” (E7-MT-18 anos).

Entre rapazes cis e heterossexuais, por sua vez, percebemos maior liberdade na aceitação de suas práticas sexuais pela família. Alguns relataram, por exemplo, que o local onde transavam era a própria casa, com a anuência da família.

“*Minha mãe sempre foi de boa, porque ela trabalhava e ficava fora de casa* (...) *Ela sabia, mas não falava nada*” (E8-M-19 anos).

“*Em minha casa* [local que costuma transar]*, quando meus pais não estão lá. Com meus pais lá, eu não me sinto à vontade*” (E12-M-15 anos).

O conteúdo da comunicação pela família pode reforçar o fenômeno da “*transgeracionalidade*”, que diz respeito aos padrões relacionais transmitidos de uma geração a outra, enfatizando estereótipos de gênero, pelos quais a mãe/mulher lida com tarefas da casa e cuidado dos filhos, e o pai/homem se ocupa da posição de provedor, reforçando desigualdades de gênero ^48^. Por outro lado, muitos familiares podem se sentir despreparados e pouco confortáveis na abordagem da saúde sexual e reprodutiva, devido à influência de normas culturais, do conservadorismo religioso e da baixa escolaridade dos pais ^47^ ^,^ ^49^ ^,^ ^50^. Além disso, os pais podem monitorar as atividades dos filhos de maneira desigual, demonstrando desigualdades de gênero no modo como realizam essa supervisão, incentivando meninos cis e reprimindo meninas cis ^35^ ^,^ ^51^.

Estudos mostram que o efeito do acesso à saúde sexual e reprodutiva abrangente pode ser positivo para modificação de comportamentos, atitudes e práticas no contexto da prevenção de IST e da contracepção ^52^. Desse modo, é fundamental que os(as) adolescentes tenham acesso às informações sobre saúde sexual e reprodutiva, abrangendo orientações sobre direitos, sexualidade, gênero, bem como acesso aos serviços de saúde e insumos de prevenção e contracepção ^53^.

## Conclusão

Observamos diferenças de gênero nas experiências de iniciação sexual entre meninos cis e meninas cis e trans. Apesar das meninas cis expressarem maior liberdade sexual e busca do prazer, ainda persistem preocupações com o julgamento conservador da família sobre a posição da mulher na sociedade em relação à sexualidade. Por outro lado, meninos cis pareceram vivenciar a sexualidade com menos preocupação e motivados pelo desejo sexual, apesar de se limitarem, por outro lado, pela pressão social dos pares.

Além disso, observamos que a família possuía um papel superficial no apoio dos(as) adolescentes no processo de iniciação sexual, com destaque da mãe como principal fonte de informações, a carência da figura paterna e a presença de outros atores em uma variedade de arranjos familiares. Mesmo entre aqueles(as) adolescentes que relataram conversar com os pais, observamos que as orientações sobre saúde sexual e reprodutiva eram superficiais e limitadas ao uso de preservativo e contraceptivo.

As experiências de iniciação sexual não se davam de modo sistemático, singular ou linear, mas de formas múltiplas e complexas, articuladas com a produção e reprodução de aspectos sociais mais amplos. O adiamento ou a antecipação das práticas sexuais genitais podem ser motivadas por sentimentos, curiosidade, influência dos pares ou parceiros ou mesmo por pressão social de redes de amizade.

As diferenças de gênero nas experiências de iniciação sexual identificadas podem contribuir para potencialização das vulnerabilidades e para produção de barreiras ao acesso à saúde sexual e reprodutiva. Nossos achados reiteram visões, práticas sexuais e normas de gênero já identificadas em outros estudos, especialmente entre adolescentes de camadas populares, sugerindo uma persistência significativa dessas estruturas normativas, mesmo diante das transformações culturais mais recentes. Esse cenário pode estar relacionado ao menor alcance das mudanças nas normas de gênero nesses contextos, marcados por restrições no acesso à informação e pela descontinuidade de políticas públicas voltadas à saúde sexual e reprodutiva. As redes sociais, por sua vez, configuram-se como espaços ambíguos: ao mesmo tempo em que podem reforçar valores tradicionais, também oferecem possibilidades ampliadas de expressão, construção de identidades e exercício da agência juvenil. Soma-se a isso o atual contexto sociopolítico pós-pandemia de COVID-19, atravessado pelo avanço do conservadorismo e pelo desmonte de políticas de saúde sexual e reprodutiva, o que tende a fragilizar ainda mais as estratégias de cuidado, autonomia e proteção dos direitos sexuais e reprodutivos de adolescentes e jovens.

Desse modo, é necessária a retomada de programas de saúde sexual e reprodutiva que vão além do modelo biológico tradicional, abordando questões de gênero, identidades e diversidade sexual, que podem ser incorporados ao currículo escolar e fornecer aos jovens informações sobre saúde sexual. Além disso, é importante fortalecer a participação de jovens e da família em programas que enfatizem autonomia, consentimento e habilidades de comunicação sem desigualdades de gênero para fomento de tomada de decisões informadas sobre saúde sexual e reprodutiva.

## Data Availability

Os dados de pesquisa estão disponíveis mediante solicitação ao autor de correspondência.
